# Re-evaluation of high-risk breast mammography lesions by target ultrasound and ABUS of breast non-mass-like lesions

**DOI:** 10.1186/s12880-021-00665-6

**Published:** 2021-10-26

**Authors:** Jianxing Zhang, Lishang Cai, Ling Chen, Xiyan Pang, Miao Chen, Dan Yan, Jia Liu, Liangping Luo

**Affiliations:** 1 Department of Medical Imaging Center, Jinan University First Affiliated Hospital, No. 613, Huangpu Road West, Tianhe District, Guangzhou, 510630 Guangdong Province China; 2 Department of Ultrasound, Guangzhou University of Traditional Chinese Medicine First Affiliated Hospital, No. 16, Jichang Road, Baiyun District, Guangzhou, 510403 Guangdong Province China; 3 Department of Ultrasound, 2Nd Clinical Hospital of Guangzhou Chinese Traditional Medicine College: Guangdong Provincial Hospital of Traditional Chinese Medicine, No. 111, Dade Road, Yuexiu District, Guangzhou, 510120 Guangdong Province China

**Keywords:** Breast cancer, Target ultrasonography, Non-mass-like lesion, Mammography, Automated breast ultrasonography

## Abstract

**Objective:**

The purpose of this study was to reevaluate the high-risk breast non-mass-like lesions (NMLs) in mammography (MG) by target ultrasound (US) and Automated breast ultrasonography (ABUS), and to analyze the correlation between different imaging findings and the factors influencing the classification of lesions.

**Methods:**

A total of 161 patients with 166 breast lesions were recruited in this retrospectively study. All cases were diagnosed as BI-RADS 4 or 5 by MG and as NML on ultrasound. While all NMLs underwent mammography, target US and ABUS before breast surgery or biopsy in the consistent position of breast. The imaging and pathological features of all cases were collected. All lesions were classified according to the lexion of ACR BI-RADS®.

**Results:**

There were significant differences between benign and malignant breast NML in all the features of target US and ABUS. US, especially ABUS, was superior to MG in determining the malignant breast NML. There was a significant difference in the detection rate of calcification between MG and Target US (*P* < 0.001), and there was a significant difference in the detection rate of structural distortion between ABUS and MG (*P* < 0.001).

**Conclusions:**

Target US, especially ABUS, can significantly improve the sensitivity, specificity and accuracy of the diagnosis of high-risk NMLs in MG. The features of Target US and ABUS such as blood supply, hyperechogenicity, ductal changes, peripheral changes and coronal features could be employed to predict benign and malignant lesions. The coronal features of ABUS were more sensitive than those of Target HHUS in showing structural abnormalities. Target US was less effective than MG in local micro-calcification.

## Key points

- Target US, especially ABUS, could significantly improve the sensitivity, specificity and accuracy in the diagnosis of high-risk non-mass-like lesions in MG, and reduce the misdiagnosis rate and biopsy rate.
- The features of Target US and ABUS such as blood supply, hyperechogenicity, ductal changes, peripheral changes and coronal features could be used to predict benign and malignant lesions.
- The coronal features of ABUS were more sensitive than Target US in showing structural abnormalities.
- Target US was less effective than MG in local micro-calcification, such as scattered, multiple punctate or cluster calcification.

## Introduction

Breast cancer screening and mammography (MG) were considered to be effective methods to reduce breast cancer-related mortality [1, 2], but this will lead to unnecessary biopsy [3], and at least 25% of detectable breast cancer will be missed[4], which included some cases of non-mass-like lesions (NMLs). NMLs are focal hypoechoic areas found on high-resolution breast ultrasound. Because NMLs are localized asymmetries on two orthogonal planes and have no distinct edges or shapes, they do not meet the strict criteria of "mass" as defined by BI-RADS. At present, breast ultrasound is considered as an important method to detect and characterize breast lesions[5]. ACR BIRADS defines the management of mass of the breast, but fails to provide the management of NMLs. Currently, there is no standard interpretation method for the classification of non—mass—like breast lesions. In previous studies[6], non-mass-like lesion was divided into hypo-echoic areas of breast, hypo-echoic areas with micro-calcification, and solid echoes in the duct. In 2004, the breast and thyroid nosology Association of Japan systematically organized and classified NML. Their criteria included abnormal ductal changes, multi-vesicular patterns, hypo-echoic areas of breast tissue, and structural aberrations[7].

According to the lexion of ACR MG-BI-RADS, the category of BI-RADS 4 or 5 was breast lesions that need pathological examination. However, in clinical practice, some non-mass-lesions were pathologically diagnosed as benign lesions, which were classified as BI-RADS 4 or 5 by MG. *Kai-HsiungKoa* [8]classified breast NML into four categories followed calcification and architectural distortions, which were correlated with different BI-RADS categories. However, the overlapping features make it difficult to diagnose the nature of breast NML, which make the tissue biopsy necessary for standard identification. Thus, the features found under ultrasound and the standardized classification of NML on US(ultrasound) can help to reduce the number of unnecessary biopsies [5]. Automated breast ultrasonography (ABUS/ABVS)is a new imaging technology of ultrasound that can provide standardized image acquisition and coronal images of the entire breast. Through examining the breast continuously in transverse section, ABUS can automatically carry on the breast reconstruction in three-dimensional condition and simultaneously obtain the morphologic and coronal images [9]. In previous studies, it has been reported that ABUS can improve the sensitivity, specificity and accuracy of distinguishing the nature of breast lesions [10].

The corresponding mammography findings were micro-calcification, structural distortion, local asymmetry and ductal changes. Mammography also had high status on the display of NML image features[8]. and all of these manifestations of MG were defined by ACR BI-RADS as suspicious lesions which required to biopsy. How to use target ultrasound and ABUS to reevaluate the suspicious non-mass-like lesions of MG and reduce the risk of unnecessary biopsy is an important topic in clinical research. The purpose of this study was to reevaluate the suspicious non-mass-breast lesions in MG by target US and ABUS according to the lexion of ACR BI-RADS, and to analyze the correlation between different imaging findings and the factors influencing the classification of lesions. High-risk NML was reevaluated in order to explore more valuable diagnostic indicators for screening and to reduce the misdiagnosis rate and biopsy rate.

## Materials and methods

### Patients

The study was carried out in accordance with the Declaration of Helsinki and was approved by the local Institutional Review Board (ZE2020-232).

A total of 161 patients (aged from 24 to 68) with 166 breast lesions between April,2017 to December,2019 were included in this retrospectively study. All cases were diagnosed with breast NML by US. In this study, all NMLs were diagnosed as BI-RADS 4 or 5 by MG. Among them, 14 cases (with high-risk of family or personal history of cancer) were younger than 35 years old. All NMLs underwent mammography, target US and ABUS before breast surgery or biopsy in the consistent position of breast.

**Fig. 1:**
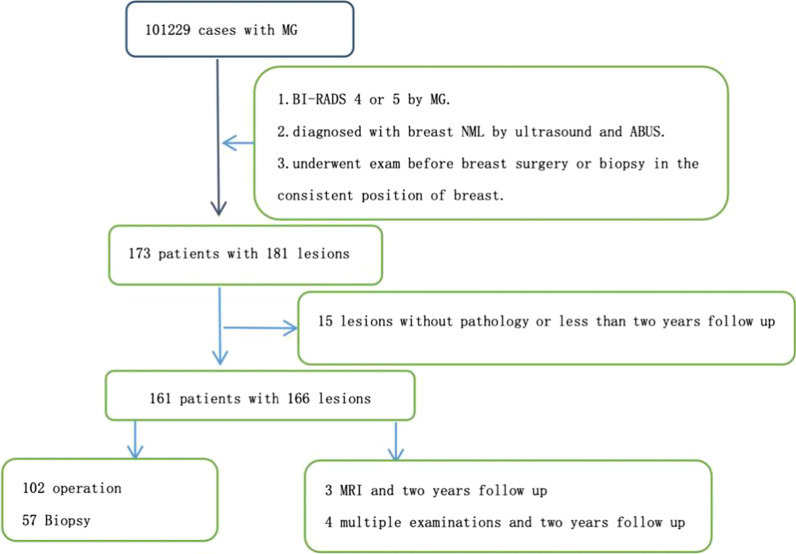
The flow chart depicting the inclusion and exclusion of patients

159 cases were completed parallel by surgery and pathological examinations within six months.7 cases of breast NML were considered to be benign by breast MRI (1 cases were diagnosed as BI-RADS category 1 by MRI, 2 cases were diagnosed as BI-RADS category 3 by MRI) or multiple reexaminations (4 cases were diagnosed as BI-RADS category 3 by ultrasound and ABUS). All those 7 cases were found no change after 2 years follow-up by multiple reexaminations. Patients without all the above examinations were excluded of the study. Also, patient who had undergone pathological or surgical examination before the exam was not conforming to the criterion. The flow chart of the included subjects was shown in Fig. 1.

### Imaging analysis

All examinations of target US and ABUS were performed with GE logiq E9 (GE) with ML6-15 liner probe in 10-14 MHz, I900 (CANON) with i18LX5 liner probe in 10-16MHzand GE invenia ABUS (GE) with C15-6XW arc probe in 10 MHz. Central, medial and lateral scans are performed on each side of the breast, with upper and lower azimuth scans added if necessary. After the scan, images were stored with breast location markers and transmitted to the workstation for automatic three-dimensional reconstruction. The transverse, sagittal and coronal images of the breast were analyzed. The mammography (MG) features were obtained using Hologic-Lorad M-IV. All images are stored in the PACS system and subjected to a fuzzy preprocessing to avoid the impact of uncertainty and / or inaccuracy[11]**.** The MG examinations were performed by two radiologists with above 15y of experience in breast images. The target US and ABUS examinations were performed by two experienced breast sonographers with 15y or 22y experience in breast US and 4 years of experience in ABUS.

**Fig. 2:**
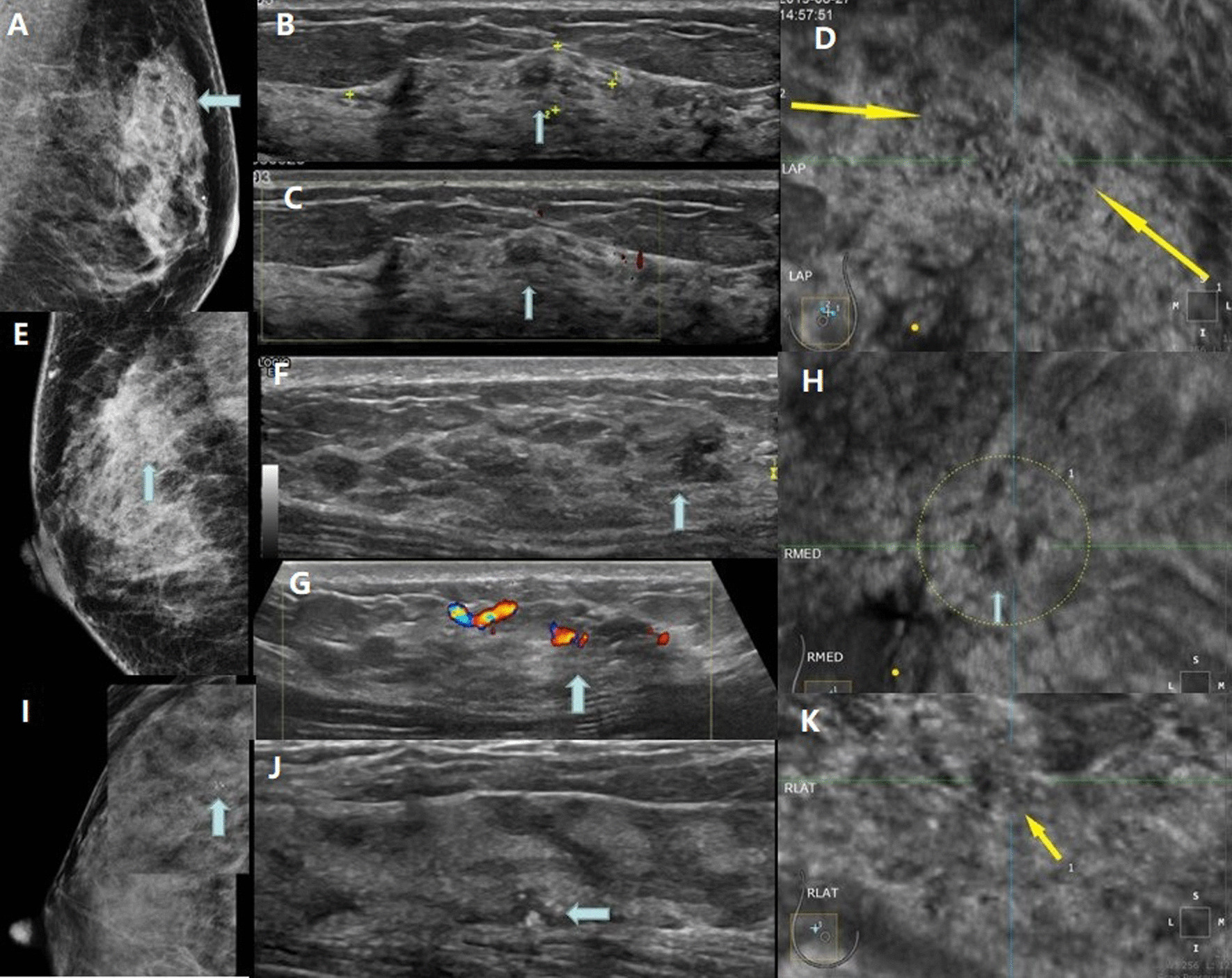
**A**-**D** 46y, female. **A**: Patchy distribution of amorphous calcification in the upper quadrant of the left breast. MG diagnosis: BI-RADS 4B. **B** There was a patchy hypoechoic area in the upper quadrant of the left breast, in which there were scattered hyperechoic spots along the catheter, **C** and there was no obvious blood flow signal in. Ultrasound diagnosis: BI-RADS 4A. **D** On the coronal plane of ABUS, there was a focal heterogeneous echo area with heterogeneous internal echo and patchy hypoechoic edge.ABUS diagnosis: BI-RADS 4A. Pathology:Fibrocystic breast disease in the left breast with extensive intraductal calcification, flat foci and atypical hyperplasia of epithelium. **E**–**H**: 48 y, female. **E** Patchy high density shadow can be seen in the right supra region, and there is no obvious calcificationin it. Diagnosis of MG: BI-RADS 4A. **F** There was a low heterogeneous echo area 12 mm away from the nipple at 12 points of the right breast. There was no clear boundary with the surrounding tissue, and there was no dot hyperecho in the area. **G** CDFI showed a slight increase of blood flow signal in heterogeneous echo area, Adler grade 2. Ultrasound diagnosis: BI-RADS 4B. **H** There were heterogeneous echo areas in the coronal plane of ABUS, with uneven internal echo and uneven coronal edge. Diagnosis: BI-RADS 4B. Pathology:Breast intraductal carcinoma. **I**-**k** 28 y, female. **I** Mammography found focal cluster calcification in the upper quadrant of right breast, MG: BI-RADS 4B. **J** In the second eye of this lesion, multiple microcalcifications of 5 mm in the right breast were found by ultrasound, with low echo around them. US: BI-RADS 4B. **K** ABUS showed focal patchy heterogeneous echo area with several focal hyperechoic spots, fuzzy edges and no convergence sign. ABUS: BI-RADS 4B. Pathology: breast intraductal carcinoma

As the current lexion of ACR BI-RADS covers only mass lesions, there is no standardized NML classification method [12]. In this study, masses were classified according to the lexion of ACR BI-RADS [12]. The vascularity of the NML in color Doppler mode was classified on the basis of Adler’s grade into four categories**.** All the features of breast NML in mammography or MRI were evaluated with the lexion of ACR BI-RADS®(Fig. 2). NML mostly exhibited ill-defined margins, irregular shape and unparallel orientation. Since not all patients exhibit the typical features of breast NML, any suspicious feature was recorded on MG examination. Based on the MG results, HHUS and ABUS were performed to evaluate the sonographic characteristics of NML and to focus on the lesions. All features of breast NML were evaluated and recorded, including location, maximum diameter, echo pattern, structural distortion, ductal changes, microcalcification (hyperechoic < 2 mm in diameter[13]) and posterior echo.

Accorded to the guideline of ACR BI-RADS®, the other group was recommended pathological examination when the BI-RADS category were 4A to 5, and the group was recommended follow-up when the BI-RADS category were 2 to 3. The higher the BI-RADS grade, the higher the probability of malignant pathological diagnosis. The grades lower than 4A were considered as the benign group, the grades higher than 4B were defined as malignant group. So we also analyzed the groups as suspicious benign when BI-RADS category was 1 to 4A and suspicious malignant group when BI-RADS category was 4B to 5.

### Establishment of the final diagnosis

The final diagnoses were based on pathological results or follow-up by ultrasonography for more than 2 years. Pathological results, whether obtained by US-guided core needle or excisional biopsy, were considered definitive. In our study, total 159 cases were completed pathological examinations by surgery. According to the 2019 WHO classification of tumor of the breast [14]**,** DCIS(Ductal carcinoma in situ) and LCIS(Lobular carcinoma in situ) were defined as uncertain malignant potential (B3). The breast malignant tumors were defined as malignant lesions (B5). Atypical ductal hyperplasia (ADH), flat epithelial atypia (FEA), and other types of benign lesions were included in the benign lesions group (B2).

### Statistical analysis

All quantitative data with normal distribution and homogeneity of variance were expressed as mean ± standard using t-test while non-normal distributions or variances using *Kruskal–Wallis* Test. Categorical data was expressed by composition ratio or rate ratio, and the differences between groups were compared using *chi-square* test or *Fisher's Exact Test*, and the influencing factors were analyzed by unconditional stepwise logistic regression. The sensitivity, specificity and accuracy of MG, HHUS and ABUS were calculated for differentiating breast NML using the final pathologic findings as a reference. All data was analyzed using SPSS 22.0 (Chicago, USA). *P* < 0.05 was considered to indicate a significant difference.

## Results

### Pathologic diagnosis of breast NML

Among the 166 patients, 29 (17.57%) were malignant (B5), 33 (19.88%) were malignant potential (B3) and 104 (62.7%) were benign (B2). The pathologic diagnoses were listed in Table 1.

**Table 1:** Pathological results of the breast NML

	Pathological diagnosis	Number(%)
Malignant or precursor		N = 62 (100)
	Invasive carcinoma (non-special type)(B5)	27 (43.5)
	Multiform lobular carcinoma(B5)	1 (1.6)
	multiform invasive lobular carcinoma(B5)	1 (1.6)
	ductal carcinoma(B3)	30 (48.4)
	Solid papillary carcinoma(B3)	3 (4.8)
Benign	(B2)	N = 104 (100)
	Hyperplasia without calcification	18 (17.3)
	Hyperplasia with calcification	23 (22.1)
	Sclerosing adenosis	5 (4.8)
	Sclerosing adenosis with calcification	8 (7.7)
	Radial scar with calcification	1 (1.0)
	Papilloma	7 (6.7)
	Papilloma with calcification	2 (1.9)
	Adenomatous hyperplasia	5 (4.8)
	Adenomatous hyperplasia with calcification	6 (5.8)
	Stromal pseudoangiomatous hyperplasia	1(1.0)
	Ibroadenoma with calcification	1 (1.0)
	Adenosis with infection and calcification	1 (1.0)
	Hyperplasia with apocrine metaplasia and calcification	4 (3.8)
	ADH	2 (1.9)
	ADH with calcification	5 (4.8)
	FEA	3 (2.9)
	FEA with calcification	3 (2.9)
	ADH with papilloma and calcification	2 (0.2)
	Benign after 2-years follow-up	7 (6.7)

### Association between imaging features and pathological diagnosis

In this study, the comparison of imaging features of MG and pathological diagnosis was shown in Table 2.

**Table 2:** Imaging features of MG, ultrasound (HHUS and ABUS) and pathological diagnosis

		Total (n = 166)	Pathologically benign(n = 104)	Pathologically malignant or precursor (n = 62)	$\chi^{2}$	*P*
Structure distortion X-ray	Non	102	71	31	6.074	0.048
	Distortion	32	15	17		
	Disorder	32	18	14		
Local thickening	Non	143	97	46	11.842	0.001
	Thickness	23	7	16		
Calcification	Non	24	21	3	7.688	0.021
	Scattered spotty	83	47	36		
	Multiple spotty or cluster	59	36	23		
Internal echo	Homogeneous	13	10	3	Fisher	0.375
	Inhomogeneous	153	94	59		
Hyperechoic	Non	63	47	16	8.708	0.013
	Scattered spotty	54	26	28		
	Multiple spotty or cluster	49	31	18		
Peripheral flow	Adler 0	97	79	18	50.092	< 0.001
	Adler 1	34	20	14		
	Adler 2	19	2	17		
	Adler 3	16	3	13		
Internal flow	Adler 0	95	79	16	50.665	< 0.001
	Adler 1	35	19	16		
	Adler 2	19	3	16		
	Adler 3	17	3	14		
Ductal change	Non	120	76	44	12.825	0.002
	Duct ectasia(anechoic inside)	17	16	1		
	Duct ectasia(Low-echo inside)	29	12	17		
Peripheral change	Non	137	97	40	24.162	< 0.001
	Structure distortion	28	6	22		
Coronal plane	Non	65	61	4	56.489	< 0.001
	Obscure	63	35	28		
	Distortion	38	8	30		

HHUS and ABUS were used to analyze the imaging features of all high-risk NML lesions in MG. As shown in Table 2, there are significant differences between benign and malignant breast NML in hyper-echoic, peripheral changes, ductal changes, micro-calcification and posterior echo, peripheral flow, internal flow, and coronal features of ABUS, which indicates that the imaging features obtained by different methods are of great value for the reevaluation of breast NML.

### Diagnostic capability of different imaging techniques to determine the malignant breast NML

As shown in Table 3, MG, target US and ABUS all showed significant differences in determining the malignant breast NML (*P* < 0.05). The sensitivity in evaluating the necessity of pathological examination were 96.8%(ABUS),91.9%(target US) and 75.8%(MG). Specificity was 75.0%(ABUS), 55.8%(target US)and 42.2%(MG). Positive predictive value(PPV) was 69.8%(ABUS), 55.3%(target US) and 44.8%(MG). Negative predictive value(NPV) was 97.5%(ABUS), 92.1%(target US) and 75.4%(MG). Accuracy was 83.1%(ABUS), 69.9%(target US) and 56.0%(MG). For ABUS, the sensitivity, specificity, PPV, NPV and accuracy in determining the malignant breast NML were superior to MG and target US. Compared with MG, target US has obvious advantages in the diagnosis of breast malignant NML.

**Table 3:** Detective effect of hyperechoic on the calcification

		Total(n = 166)	Calcification(MG)			Fisher exact	*P*
			Non	Scattered spotty	Multiple spotty or cluster		
Hyperechoic	Non	63	23	20	20	49.732	< 0.001
	Scattered spotty	54	0	40	14		
	Multiple spotty	49	1	23	25		

### Detective effect of hyperechoic on the calcification

As shown in Table 4, there was significantly difference in the detective effect of ultrasound hyperechoic on the calcification by mammography (*Fisher exact value* = 49.73, *P* < 0.001), indicating that ultrasound imaging might only reveal partial calcification such as scattered spotty or multiple spotty or cluster with low efficiency.

**Table 4:** Detective effect of hyperechoic on the calcification

		Total(n = 166)	Calcification(MG)			Fisher exact	*P*
			Non	Scattered spotty	Multiple spotty or cluster		
Hyperechoic	Non	63	23	20	20	49.732	< 0.001
	Scattered spotty	54	0	40	14		
	Multiple spotty	49	1	23	25		

### Consistency of structural distortion in MG and ABUS

As shown in Table 5, there was significantly difference in the detective effect of Coronal feature of ABUS on the structural distortion by mammography ($\chi^{2}$ = 23.27, *P* < 0.001), the coronal feature of ABUS showed more structural abnormalities than MG. This indicated that Coronal feature of ABUS might reveal the structural abnormalities more sensitively than MG.

**Table 5:** Consistency of structural distortion in MG and ABUS

		Total(n = 166)	Coronal plane(ABUS)			$\chi^{2}$	*P*
			Non	Obscure	Distortion		
Structure distortion (MG)	Non	102	49	42	11	23.266	< 0.001
	Distortion	32	7	11	14		
	Disorder	32	9	10	13		

## Discussion

The importance of mammography screening has been widely recognized[15], and the early detection of breast cancer is associated with better outcomes[16, 17]. The limitations of mammography screening include over-diagnosis[18], overtreatment and false-positive rates with associated negative psychological impact[19, 20] and unnecessary costs and biopsies[15]. In this study, all cases of NML were diagnosed as high-risk cases by MG, and pathological examination was recommended, but only 62 (37.3%) cases were malignant or malignat potential (B5 or B3). As we all know, there is currently no standardized NML classification method [12]. All lesions with NML were initially classified by the lesion of ACR BI-RADS®. According to the results of reevaluation, target US, especially ABUS, could significantly improve the sensitivity, specificity and accuracy of diagnosis and reduce the misdiagnosis rate and biopsy rate in NML. The features of target US and ABUS such as blood supply, peripheral change, coronal feature of the lesion had diagnostic value in the prediction of benign and malignant lesions. To our knowledge, there have been no study on reevaluation of high-risk NML under MG using by target US and ABUS.

Although the classified method of *Kai-Hsiung Koa* was effective in previous study [8], this method is not the standardized method for categorization of NML and cannot cover all the NML such as single duct dilatation and focal micro-calcification. It is necessary to further study the basic image feature elements of NM. Different imaging could provide different morphological evaluation information, which included the micro-calcification, asymmetry and structural distortion of mammography [21]**,** hyper-echo, blood supply, peripheral change of HHUS, and coronal feature of ABUS. The basic objective of mammography is to identify densities, micro-calcification, and asymmetry [22]**.** Micro-calcification may be focal or diffuse. Multifocal and small-calcification are more likely to be malignant, while homogeneous and large-calcification are usually benign. They may be stable or change overtime[23]. Micro-calcification in MG showed dot hyper-echo in ultrasound (HHUS or ABUS. Because of the different probe frequency, HHUS has higher detail resolution than ABUS in display micro-calcification). In this study, there was a statistical difference between the dot hyper-echo in ultrasound and the micro-calcification in mammography(*P* < 0.001)0.40 cases of MG showed scattered or multiple spotty or cluster calcification, which were not identified by ultrasound. In one case, multiple spots were found on ultrasound, but no calcification was found in MG. The detection of calcification in MG was consistent with pathology. This indicated that ultrasound imaging might only reveal partial calcification such as scattered spotty, multiple spotty or cluster with low efficiency.

Unlike breast masses, NMLs do not have circumscribed margins, which is an important characteristic for differentiation of breast masses. Architectural distortion and ductal changes are also common features of NMLs [24]. The pathological diagnosis of tissue structure distortion was biopsy scar, fibrosis following neoadjuvant chemotherapy, sclerosing adenosis, invasive ductal carcinoma, and DCIS [25]. In this study, structural distortion were also found in Multiform lobular carcinoma, solid papillary carcinoma, stromal pseudoangiomatous hyperplasia and so on. Advances in breast US equipment with high-frequency probes can reveal architectural distortion even in the absence of a definitive mass[25]. Structural distortion identified by US is not uncommon because of improvements in US technology. Moreover, due to the limitation of two-dimensional observation plane, only 22 malignant cases(34.5%) were found with abnormal structure. The advantages of ABUS are that it allows a non-invasive imaging of tissue distribution by realtime ultrasound with high sensitivity, specificity and accuracy[26]. In different strain levels, ABUS acquires different sonographic features of fat, normal glandular tissue, fibrous tissue, ductal carcinoma in situ and infiltrating ductal carcinoma of the breast [27]. More NMLs could detected architectural distortion by ABUS(102 cases,61.4%) than that by MG(64 cases,38.6%)(*P* < 0.001). This indicated that Coronal plane of ABUS imaging might reveal the structural abnormalities more sensitively than MG.

Vascular distribution is another important factor in differentiation diagnosis of NML, and CDFI could provide information of NML blood supply [28]. There were statistical differences in blood supply between benign and malignant lesions(*P* < 0.001). Malignant lesions showed more abundant blood supply, but there were also some cases with less abundant blood supply as malignant lesions and some benign cases with abundant blood supply. DCIS usually shows low vascularity [28], and the group of inflammatory cases also affected the judgment of benign and malignant lesions. As a result of the using wall filter to suppress clutter and motion artifacts, as well as angle dependent, low-speed signals from micro vessels will be missed, but there are still statistical differences in the evaluation of benign and malignant lesions by blood flow signals in this study(*P* < 0.001). Findings with angiographic features may be more useful for the evaluation of non-mass-like US findings.

Hypo-echoic area in the mammary gland was the most frequent sign of NMLs. Some studies have classified “hypo-echoic area” as ductal and non-ductal hypo-echoic area, according to whether the pattern they describe is reminiscent the milk duct system or those reminiscent of glandular tissue [8]. All cases of NML were diagnosed as high-risk cases by MG, there was no significant difference in hypo-echoic areas in this study.

Our research has limitations. First, differences between observers are well-known limitations of image. In order to reduce these limitations, two experienced breast imaging experts discussed the concise definition of descriptors, and reached a consensus on these definitions by using detailed documents in the retrospective analysis of target US and ABUS findings. However, the current NML definition was still need to be standardized. Secondly, the complexity of NML pathological results and a small number of cases also affect the wide representation of the results. Third, the study was conducted in a single institution with only a few cases. More research needs to be done in multiple research institutions. Fourth, the factors affecting the imaging performance of NMLs had not been explored in this study. Further research is needed to explore and prove.

In conclusion, target US, especially ABUS, can significantly improve the sensitivity, specificity and accuracy of the diagnosis of high-risk non-mass-like lesions in MG, and reduce the misdiagnosis rate and biopsy rate. The features of target US and ABUS such as blood supply, hyperechogenicity, ductal changes, peripheral changes and coronal features could be used to predict benign and malignant lesions. This indicated that the coronal features of ABUS were more sensitive than target US in showing structural abnormalities. Target US was less effective than MG in local micro-calcification.

## Data Availability

The datasets used and/or analysed during the current study are available from the corresponding author on reasonable request.

## Abbreviations

NML: Non-mass-like lesions
MG: Mammography
US: Ultrasound
ABUS: Automated breast ultrasonography
MRI: Magnetic resonance imaging
DCIS: Ductal carcinoma in situ
LCIS: Lobular carcinoma in situ
ADH: Atypical ductal hyperplasia
FEA: Flat epithelial atypia
BI-RADS: Breast imaging reporting and data system
ACR: American College of Radiology
